# Use of chlorisondamine to assess the neurogenic contribution to blood pressure in mice: An evaluation of method

**DOI:** 10.14814/phy2.14753

**Published:** 2021-02-15

**Authors:** Lucas AC. Souza, Silvana G. Cooper, Caleb J. Worker, Pratish Thakore, Yumei Feng Earley

**Affiliations:** ^1^ Departments of Pharmacology and Physiology & Cell Biology School of Medicine University of Nevada, Reno Reno NV USA; ^2^ Center for Molecular and Cellular Signaling in the Cardiovascular System University of Nevada, Reno Reno NV USA

**Keywords:** autonomic function, blood pressure, cardiac output, chlorisondamine

## Abstract

Chlorisondamine (CSD) has been used to assess the neurogenic contribution to blood pressure (BP) and vasomotor sympathetic tone in animal models. It is assumed that the reduction in BP following CSD administration is associated to decreases in cardiac output (CO) and peripheral resistance, reflecting cardiac and vasomotor sympathetic tone, respectively. Surprisingly, this has not been characterized experimentally in mice, despite the extensive use of this animal model in cardiovascular research. We hypothesize that a specific dose of CSD can selectively block the sympathetic vasomotor tone. To test this hypothesis, we evaluated the effects of different doses of CSD (intraperitoneal) on BP and heart rate (HR) using telemetry, and on CO using echocardiography. BP and HR in normotensive C57Bl/6J mice reduced to a similar extent by all CSD doses tested (1–6 mg/kg). CSD at 6 mg/kg also reduced CO without affecting left ventricular stroke volume or fractional shortening. On the other hand, lower doses of CSD (1 and 2 mg/kg) produced significantly larger BP and HR reductions in DOCA‐salt–induced hypertensive mice, indicating a greater neurogenic BP response. In addition, all doses of CSD reduced CO in hypertensive mice. Our data suggest that the BP response to CSD in mice likely reflects reduced CO and vasomotor sympathetic tone. We conclude that CSD can be used to assess the neurogenic contribution to BP in mice but may not be appropriate for specifically estimating vasomotor sympathetic tone.

## Graphical abstract

Ganglionic blocker, chlorisondamine, can be used to assess the neurogenic contribution to BP in mice, but may not be appropriate to specifically estimate the vasomotor sympathetic tone.

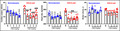

## 1 INTRODUCTION

Hypertension is one of the main risk factors for the development of cardiovascular diseases, which were responsible for more than 400,000 deaths in the United States in 2016 (Ritchey et al., 2018). Applying the new thresholds established by the ACC/AHA (Whelton et al., 2018), a recent study reported that 44% of adults are affected by hypertension (Lamprea‐Montealegre et al., 2018). The pathophysiology of hypertension is complex (Fisher & Paton, 2012; Lerman et al., 2019); however, it has been shown that sympathetic hyperactivity is a contributing factor to the development and maintenance of hypertension in humans (Fisher & Paton, 2012; Grassi et al., 2015; Mancia & Grassi, 2014) and animal models of hypertension (Lerman et al., 2019). Hence, sympathetic activity, often assessed using pharmacological approaches, is a commonly estimated parameter in studies using animal models of hypertension (Barman & Yates, 2017).

Chlorisondamine (CSD) is a nicotinic receptor antagonist and ganglionic blocker that was initially developed for the treatment of hypertension (Bakke & Darvill, 1957; Grimson et al., 1955). Initial tests were promising, showing that CSD did indeed reduce blood pressure (BP), supporting the importance of sympathetic activity in BP; however, it was not well tolerated by hypertensive subjects due to its side effects (Bakke & Darvill, 1957; Grimson et al., 1955). CSD has also been widely used by many research groups, including our laboratory, to assess autonomic function and vasomotor sympathetic tone in animal models of hypertension (Li et al., 2012, 2014, 2015; Souza et al., 2019; Xu et al., 2019; Xu, Sriramula, et al., 2017). Early studies in dogs and humans showed decreases in peripheral resistance and cardiac output (CO) following the administration of CSD, with decreases in peripheral resistance accounting for approximately 70% of the CSD‐induced reduction in BP (Trapold Joseph & Sullivan, 1956; Trapold Joseph et al., 1957). Nevertheless, it has remained unclear whether the effects of CSD on BP in mice are specifically associated with a decrease in peripheral resistance or whether decreases in CO also play a role.

We hypothesize that a specific dose of CSD can selectively block the sympathetic vasomotor tone. To test this hypothesis, here in this study, by evaluating the impact of a series of doses of CSD on BP and CO in normotensive and hypertensive mice, we examined whether a certain dose of CSD could be used to specifically assess sympathetic vasomotor tone. We found that, in general, the reduction in BP observed following the administration of CSD was associated with a bradycardic response and a decrease in CO. Thus, we conclude that CSD can be used to estimate the overall neurogenic contribution to BP in mice but is not suitable for specifically assessing sympathetic vasomotor tone.

## 2 MATERIALS AND METHODS

### 2.1 Animals and treatments

Male and female wild‐type (WT) mice (12–14 weeks old) in a C57Bl/6J background were used in this study. All mice were maintained in the animal facility at the University of Nevada, Reno, under a 12‐h light‐dark cycle and room temperature of 21–23°C with ad libitum access to a standard chow diet (0.1% sodium, catalog #2019; Envigo) and water. The effects of different doses of CSD (1, 2, 3, or 6 mg/kg) on BP and cardiac function were tested in normotensive mice and DOCA‐salt–induced hypertensive mice, the latter of which were produced by subcutaneously implanting WT mice with a 50 mg DOCA pellet (21‐day release; Innovative Research of America, Sarasota, FL). CSD solutions for in vivo studies were prepared in sterile 0.9% saline and administered by intraperitoneal (i.p.) injection on different days, allowing at least 48 hours for recovery; 0.9% saline was used as a vehicle. Mice in both normotensive and hypertensive groups were provided *ad libitum* access to standard chow and either tap water or 0.9% NaCl solution, as previously described (Li et al., 2014, 2015; Souza et al., 2019; Trebak et al., 2018). Since preliminary studies revealed no sex differences in BP or cardiac function, data from both genders were combined for all analyses performed in this study. All procedures were conducted in accordance with the National Institutes of Health Guide for the Care and Use of Laboratory Animals and were approved by the Institutional Animal Care and Use Committee at the University of Nevada, Reno.

### 2.2 Telemetric determination of BP and HR

Mice were anesthetized using 4%–5% isoflurane in 100% O_2_, flushed at 1 L/min for 2 min. Anesthesia was then maintained using 0.75–1.5% isoflurane. After the neck was shaved and sterilized with alcohol swabs, an incision (~1 cm) was made to separate the oblique and tracheal muscles and expose the left carotid artery. The catheter of a radio telemetry transmitter (PA‐C10; Data Science International, Harvard Bioscience, Inc.) was surgically implanted into the left carotid artery and secured. The body of the radio transmitter was embedded in a subcutaneous pocket under the right arm. Mice were allowed to recover for 14 days (Souza et al., 2019). Maximum changes in mean arterial pressure (MAP) and HR in response to i.p. injections of CSD were analyzed (Souza et al., 2019).

### 2.3 Echocardiographic evaluation of cardiac function

Echocardiograms (Visualsonics VEVO 2100; Fujifilm) were performed in both normotensive and DOCA‐salt hypertensive mice at baseline and 30 min after i.p. injection of CSD. Anesthesia was induced with 5% isoflurane (1 L/min) and maintained with constant 1.5% isoflurane (1 L/min). ECG electrodes were placed in a standard limb configuration for monitoring HR. Ultrasound 2D images of parasternal long and short axes of the left ventricle (LV) at the level of papillary muscles were obtained using a 40 MHz linear transducer. M‐mode echocardiographic images were analyzed using Vevo Lab software 3.1.1 (Visualsonics). Measurements of CO, LV stroke volume, ejection fraction, LV end diastolic volume (LVEDV), and fractional shortening were acquired, and measurements from long and short axes (three different cardiac cycles) of the LV for each variable were averaged.

### 2.4 Measurement of cardiac and renal hypertrophy

After 21 days of sham or DOCA‐salt treatment, mice used in the echocardiography study were euthanized by cervical dislocation. Body weight (g), heart and left kidney weight (mg), and the length of the right tibia (mm) were measured. Cardiac and renal hypertrophy were then determined by normalizing heart and kidney weights to body weight and tibia length.

### 2.5 Statistical analysis

Data are presented as means ± standard errors (SE) and were compared using Prism 9.0.0 software (GraphPad). For the comparison of BP, HR, and cardiac function and account for possible interactions between the independent variables condition (normotensive or hypertensive) and treatment with CSD (1, 2, 3, or 6 mg/kg), data were compared with two‐way ANOVA followed by the Fisher's LSD post‐hoc test. Data were checked if the data pass the assumptions to perform statistical comparisons using this test. Importantly, outliers were checked using the ROUT method and data were tested for variance homogeneity (Spearman's test) and Gaussian distribution (Kolmogorov–Smirnov test). For anthropometric measurements, data were assessed for the presence of outliers (ROUT method) and tested for variance homogeneity (*F*‐test) and Gaussian distribution (Shapiro–Wilk test). Parametric Student's *t*‐tests or nonparametric Mann–Whitney tests were used for the statistical comparisons. No data were removed in this study. A *p*‐value ≤0.05 was considered statistically significant.

## 3 RESULTS

### 3.1 CSD reduces BP and CO in normotensive mice

**FIGURE 1:**
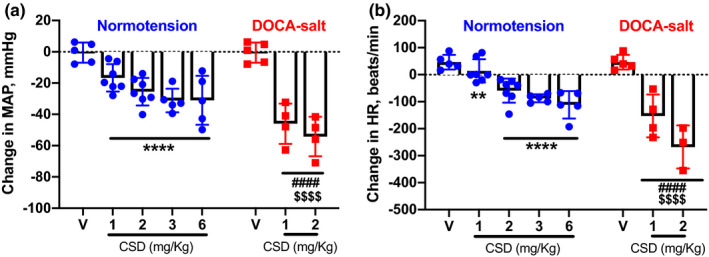
Ganglionic blockade with CSD reduces BP and HR in normotensive and hypertensive mice. Changes in MAP (a) and HR (b) in normotensive and DOCA‐salt hypertensive mice (*n* = 4–7 mice/group; **p < 0.01, ****p < 0.0001 vs. Vehicle in Normotension; ^####^ *p *< 0.0001 vs. Vehicle in DOCA‐salt; ^$$$$^ *p* < 0.001 vs. the response to the respective CSD dose in normotensive mice; two‐way ANOVA followed by Fisher's LSD post hoc test). V, vehicle

To investigate the cardiovascular effects of CSD in normotensive mice, we assessed BP and HR responses to different doses of CSD (1, 2, 3, and 6 mg/kg; i.p.) using a telemetric recording system. We found that all doses of CSD significantly reduced BP, decreasing mean arterial (ΔMAP) by 16.7 ± 3.3, 25.5 ± 3.3, 31.1 ± 3.4, and 31.0 ± 7.0 mmHg at 1, 2, 3, and 6 mg/kg, respectively, compared with vehicle (−0.49 ± 2.9 mmHg; *p *< 0.0001; Figure 1a), indicative of a sympathetic contribution to BP. CSD also significantly reduced HR in normotensive mice at 1 mg/kg (−14.3 ± 16.3 beats/min), 2 mg/kg (−59.1 ± 16.8 beats/min), 3 mg/kg (−87.5 ± 6.6 beats/min), and 6 mg/kg (−111.4 ± 22.8 beats/min) compared with vehicle (+46.4 ± 12.2 beats/min; *p* < 0.01; Figure 1b). Interestingly, changes in BP and HR were not significantly different among groups treated with different doses of CSD (Figure 1a,b).

**FIGURE 2:**
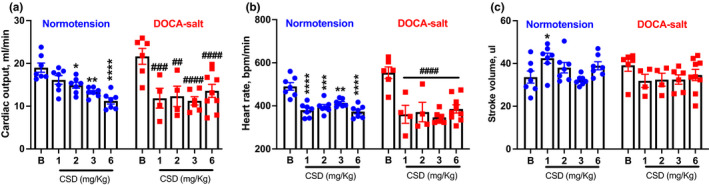
The nicotinic receptor antagonist CSD changes cardiac function in normotensive and DOCA‐salt hypertensive mice. Cardiac output (a), heart rate (b), and stroke volume (c) in normotensive and DOCA‐salt hypertensive mice (*n* = 4–9 mice/group; *p < 0.05, **p < 0.01, ***p < 0.001, ****p < 0.0001 vs. Baseline in Normotension; ^##^ *p *< 0.01, ^###^ *p *< 0.001, ^#^ *^###^p* < 0.0001 vs. Baseline in DOCA‐salt hypertension; two‐way ANOVA followed by Fisher's LSD post hoc test). B, Baseline

**FIGURE 3:**
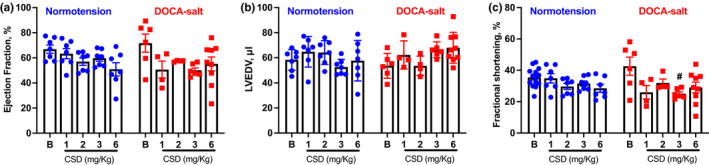
The nicotinic receptor antagonist CSD changes cardiac function in normotensive and DOCA‐salt hypertensive mice. Ejection fraction (a), LVEDV (b), fractional shortening (c) in normotensive, and DOCA‐salt hypertensive mice (*n* = 4–9 mice/group; #*p* < 0.05 vs. Baseline in DOCA‐salt hypertension;Tto‐way ANOVA followed by Fisher's LSD post hoc test). B, Baseline

We further examined the effects of CSD on cardiac function in mice using in vivo 2D‐guided M‐mode echocardiography. We found that CSD significantly reduced CO in normotensive mice only at the dose of 2, 3, and 6 mg/kg compared with baseline values (*p* < 0.01; Figure 2a). In addition, CSD significantly reduced HR in normotensive mice, reducing it to 380.3 ± 11.7 beats/min at 1 mg/kg, 395.5 ± 10.9 beats/min at 2 mg/kg, and 373.3 ± 10.9 beats/min at 6 mg/kg, when compared with the baseline (*p* < 0.05; Figure 2b). Interestingly, there was a significant higher stroke volume when normotensive mice were treated with 1 mg/kg of CSD, which was related to no reduction in CO in this dose (Figure 2a). There was no change in stroke volume following all other doses of CSD administration in normotensive mice when compared to baseline (Figure 2c). Ejection fraction, LVEDV, or fractional shortening (Figure 3a–c) were similar among baseline and any dose of CSD. The data suggest that CSD‐induced reductions in BP in normotensive mice are associated with reduction in cardiac output.

### 3.2 CSD decreases BP and CO in DOCA‐salt hypertensive mice

In hypertension, increased BP is associated with increased sympathetic tone to the heart and blood vessels (Fisher & Paton, 2012; Lerman et al., 2019). Here, we used the DOCA‐salt–induced hypertension model, which exhibits a well‐characterized increase in sympathetic activity (Lerman et al., 2019; Li et al., 2014; Souza et al., 2019), in conjunction with a radiotelemetry system to examine BP and HR responses to CSD. We found that CSD significantly decreased BP and HR in hypertensive mice. CSD decreased MAP by 46.0 ± 6.5 and 54.2 ± 6.3 mmHg at doses of 1 and 2 mg/kg, respectively, compared with i.p. injection of vehicle (ΔMAP: −0.5 ± 2.9 mmHg; *p* < 0.0001), and producing corresponding reductions in HR of 152.7 ± 39.6 beats/min and 197.5 ± 77.5 beats/min versus a ΔHR of +46.4 ± 12.2 beats/min in vehicle‐injected (i.p.) mice (*p* < 0.01; Figure 1). There was no significant difference in BP or HR responses between 1‐ and 2‐mg/kg doses of CSD (Figure 1). It is important to highlight that BP and HR reductions following the administration of the same doses of CSD (1 and 2 mg/kg) were significantly larger in DOCA‐salt hypertensive mice when compared to normotensive mice (Figure 1).

To investigate the effects of CSD administration on cardiac function in hypertensive mice, we again used echocardiography. We found that CSD significantly decreased CO in DOCA‐salt hypertensive mice at all doses, reducing it to 11.9 ± 2.3 ml/min at 1 mg/kg, 12.3 ± 2.4 ml/min at 2 mg/kg, 11.2 ± 1.0 ml/min at 3 mg/kg, and 13.6 ± 1.5 ml/min at 6 mg/kg compared with a baseline value of 21.6 ± 1.9 ml/min (*p* < 0.01; Figure 2A). These results confirm that ganglionic blockade with CSD changes cardiac output in hypertensive mice. In addition, CSD at the doses of 1 and 2 mg/kg significantly reduced HR in DOCA‐salt hypertensive mice, reducing it to 361.0 ± 41.6 beats/min at 1 mg/kg, 371.3 ± 45.2 beats/min at 2 mg/kg, 346.9 ± 11.7 beats/min at 3 mg/kg, and 385.7 ± 19.1 beats/min at 6 mg/kg when compared with a baseline value of 553.8 ± 27.1 beats/min (*p* < 0.0001; Figure 2b). There was no significant difference in stroke volume (Figure 2c) or ejection fraction (Figure 3a) at any dose of CSD due to data variability, although both parameters trended lower. There was no difference in LVEDV (Figure 3b) at any dose of CSD. Fractional shortening exhibited a similar tendency toward a reduction with different doses of CSD that was significant only at a dose of 3 mg/kg (25.1% ± 1.2%) compared with baseline (42.6% ±5.8%; *p *< 0.05; Figure 3c).

**FIGURE 4:**
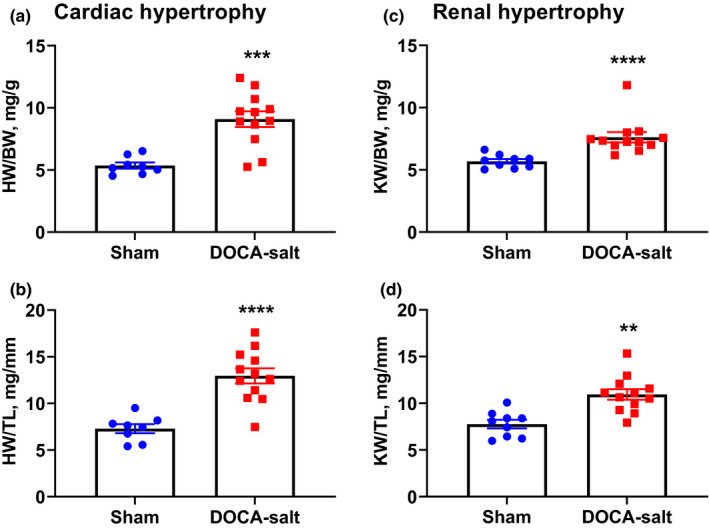
DOCA‐salt hypertensive mice develop cardiac and renal hypertrophy. Heart and kidney weight, normalized to body weight (a, c), and tibia length (b, d), as measures of cardiac (a, b) and renal (c, d) hypertrophy. Sham, *n* = 8–9 mice/group; DOCA‐salt, *n* = 12 mice/group; **p < 0.01, ***p < 0.001, ****p < 0.0001 vs. Sham. (b, d) Unpaired *t*‐test; (a, c) Mann–Whitney test

To confirm that mice used for echocardiography were hypertensive, at the end of the echocardiography study, we determined ratios of heart weight (HW) and kidney weight (KW) to body weight (BW) or tibia length (TL). As expected, DOCA‐salt hypertensive mice developed cardiac (Figure 4a,b) and renal (Figure 4c,d) hypertrophy.

## 4 DISCUSSION

Autonomic dysfunction and sympathetic hyperactivity have been shown to play important roles in the development and maintenance of hypertension in both humans (Esler et al., 1991; Grassi et al., 2015; Mancia & Grassi, 2014) and animal models of hypertension (Basting & Lazartigues, 2017; Lerman et al., 2019; Li et al., 2014; Souza et al., 2019). Pharmacological approaches for assessing sympathetic tone in chronic experiments have been widely used because they can be performed at different time points in conscious freely moving mice (Barman & Yates, 2017). Effects of ganglionic blockade on BP have been reported in different species, including humans (Grimson et al., 1955; Page & Olmsted, 1963), dogs (Grimson et al., 1955; Maxwell et al., 1956; Trapold Joseph & Sullivan, 1956; Trapold Joseph et al., 1957), rats (Santajuliana et al., 1996), mice (Li et al., 2012, 2014, 2015; Lu et al., 2009; Souza et al., 2019; Xu et al., 2019; Xu, Sriramula, et al., 2017), and pigeons (Chadman & Woods, 2004). In particular, CSD has been used by different groups to assess autonomic function (Marina et al., 2020; Nomura et al., 2019; Xu, Lu, et al., 2017; Xu et al., 2019) or as part of an attempt to distinguish between the overall sympathetic contribution to BP and sympathetic activity toward blood vessels, called sympathetic vasomotor tone (Cerutti et al., 1994; Li et al., 2012, 2014, 2015; Lu et al., 2009; Sabharwal et al., 2016; Souza et al., 2019). Surprisingly, whether the BP reductions observed following administration of CSD are a consequence of a decrease in sympathetic vasomotor tone specifically or a decrease in the overall sympathetic tone has not been characterized experimentally in mice. We report here that, in normotensive mice, most doses of CSD reduced BP and HR, while in hypertensive mice, even doses of CSD as low as 1 mg/kg reduced BP, HR, and CO. Since CO is determined by both HR and stroke volume and we did not observe significant changes in stroke volume following the administration of CSD, it is likely that changes in CO were associated mostly with a negative chronotropic effect (HR) rather than an inotropic effect (stroke volume) on the heart. These data suggest that CSD is appropriate for use in assessing the overall neurogenic contribution to BP in mice, but may not be accurate for specifically assessing vasomotor sympathetic tone.

In this study, we observed similar reductions in BP with administration of different doses of CSD in both normotensive and hypertensive mice, confirming comparable results on normotensive animals reported in previous studies (Chadman & Woods, 2004; Santajuliana et al., 1996). In addition, we found that CSD produced a peak BP response of approximately 20–30 mmHg in normotensive mice and ~55 mmHg in hypertensive mice. These results suggest a larger contribution of the sympathetic nervous system to the maintenance of BP in hypertension, as previously shown by our group and others in Ang II (Lerman et al., 2019; Li et al., 2012; Nunes & Braga, 2011) and DOCA‐salt (Banek et al., 2019; Basting & Lazartigues, 2017; Li et al., 2014, 2015; Souza et al., 2019; Xu et al., 2019; Xu, Sriramula, et al., 2017) hypertensive mouse models. Indeed, it is well characterized in the literature that the neurogenic component plays a key role in the development and maintenance of DOCA‐salt hypertension (Basting & Lazartigues, 2017; Lerman et al., 2019).

We further found that, in awake freely moving mice, CSD administration not only reduced BP but also resulted in a bradycardic response in both normotensive and hypertensive mice. The reductions in HR in hypertensive mice are significantly larger when compared with normotensive mice. However, there was no difference in the HR response to CSD when different doses of CSD were compared either among the normotensive or hypertensive mice. Early studies from the 1950s showed that CSD decreases BP through reductions in total peripheral resistance and cardiac output in dogs (Trapold Joseph & Sullivan, 1956; Trapold Joseph et al., 1957). In rats, Santajuliana and colleagues (Santajuliana et al., 1996) compared the effect of three ganglionic blockers (CSD, hexamethonium, and trimethaphan) on HR and reported a bradycardic response in normotensive rats. This bradycardic response to the administration of ganglionic blockers has also been demonstrated by other studies (Huang et al., 1994; Nakata et al., 1991). Since decreases in HR usually lead to a reduction in CO, and both CO and peripheral resistance are determinants of BP (Mayet & Hughes, 2003), these previous observations, taken together with our current results, suggest that the reductions in BP induced by CSD may be caused by a combination of decreased vasomotor and cardiac sympathetic tone. However, to our knowledge, the effect of CSD on cardiac function or cardiac output in mice have not been previously examined.

To investigate whether the effects of CSD on BP are associated with a decrease in CO in mice, we evaluated cardiac function using echocardiography. We found that administration of CSD reduced CO in both normotensive and DOCA‐salt hypertensive mice, an effect that was associated with a bradycardic response of the heart. In agreement with the results of our study, Trapold Joseph et al. (1957), using an open‐chest preparation with a cannulated heart, found that CSD (0.3 mg/kg) reduced both CO and peripheral resistance in dogs. In their study, the decrease in peripheral resistance accounted for ~70% of the decrease in BP, whereas the effect of CO (30%) was associated with a decrease in venous return to the heart. In another study in awake and cannulated dogs, Page and Olmsted (1963) tested the effect of intravenous injection of CSD (1 mg/kg) and reported that most dogs exhibited a drop in BP, CO, SV, and peripheral resistance in association with an increase in HR after the administration of CSD. Taken together, the results from the current study and previous studies suggest that reductions in CO may play a role in the BP reductions that follow the administration of CSD.

The results of our study regarding BP, HR, and CO in mice generally align well with previous results obtained in other species. We observed consistent reductions in HR with the administration of CSD in both normotensive and hypertensive mice. Interestingly, lower doses of CSD (1 mg/kg) reduced HR but not CO in normotensive mice, whereas the lower doses of CSD significantly reduced both HR and CO in hypertensive mice. Collectively, these results suggest that hypertensive mice respond more strongly or are more sensitive to ganglionic blockade—an indication of elevated sympathetic tone (Basting & Lazartigues, 2017; Li et al., 2012, 2014, 2015; Souza et al., 2019). Although no study to date investigates if the affinity to CSD is different in hypertensive mice versus normotensive mice, we could not rule out this possibility that might play a role in the difference in dose–response observed between normotensive and hypertensive mice in our study. We did not observe statistically significant reductions in stroke volume and contractility (ejection fraction and fractional shortening) for all the CSD doses tested in this study; however, we note that there was a tendency toward a reduction in contractility with the higher dose of CSD in normotensive mice and with all CSD doses in hypertensive mice. One reason we might have not observed differences in stroke volume and cardiac contractility following the administration of CSD could be that cardiac function was assessed after 30 min of CSD administration. In this condition, it is possible that compensatory humoral mechanisms activated by BP reductions, like the increase in Ang II (Stocker et al., 2000) and vasopressin (Ohman et al., 1990; Schiltz et al., 1997) levels, might have masked the effects of CSD on cardiac contractility and LV preload. Nevertheless, from these data, we summarize that the major contribution for the decrease in cardiac output is probably the reduction in heart rate in response to CSD.

A limitation of this study is that BP and HR responses to CSD were assessed in awake freely moving mice using a telemetric system, whereas the CO response to CSD was evaluated in anesthetized mice using echocardiography. Currently, there is no approach available for evaluating CO in conscious freely moving mice. However, although isoflurane has been reported to alter autonomic function and BP (Constantinides et al., 2011; Seagard et al., 1983), it is important to highlight that, in the current study, CO was measured under anesthesia at both baseline and after the administration of CSD; thus, we were able to isolate the effect of CSD on cardiac function. In addition, similar reductions in HR in response to the administration of CSD were observed in both awake and anesthetized mice, indicating a consistent bradycardic response to CSD that was not affected by isoflurane. A second limitation of this study is that we did not monitor the BP of DOCA‐salt–treated hypertensive mice used for the echocardiography study so as to avoid ligation of the carotid artery and associated surgical impacts on cardiac function. However, this is a well‐established animal model of hypertension in our laboratory (Li et al., 2014, 2015; Souza et al., 2019; Trebak et al., 2018) and we are confident that these mice developed hypertension. Indeed, to confirm that these mice developed hypertension, we measured heart weight‐to‐tibia length and kidney weight‐to‐tibia length ratios, a commonly used method for estimating cardiac and renal hypertrophy (Thibodeau et al., 2014; Yin et al., 1982) induced by hypertension.

In summary, we report here that the ganglionic blocker CSD decreases BP and HR in association with a reduction in cardiac output, suggesting that the BP response to CSD is a combination of blockade of cardiac and vasomotor sympathetic tone in both normotensive and hypertensive mice. We conclude that CSD is appropriate for use in assessing the overall neurogenic contribution to BP in mice but may not be sufficiently accurate to assess vasomotor sympathetic tone. We propose that a ganglionic blocker that can reduce BP without affecting heart rate would be a better reagent should sympathetic vasomotor tone be the interest of investigation.

## CONFLICT OF INTEREST

The authors declare that there are no competing interests associated with the manuscript.

## AUTHOR CONTRIBUTIONS

L.A.C.S. and Y.F.E. conceived and designed the research; L.A.C.S. and S.G.C. performed experiments; L.A.C.S., S.G.C., C.J.W., P.T., and Y.F.E. analyzed data; L.A.C.S., S.G.C., C.J.W., P.T., and Y.F.E. interpreted experimental results; L.A.C.S. and Y.F.E. prepared figures; L.A.C.S. and Y.F.E. drafted the manuscript; L.A.C.S., C.J.W., W.L., F.T., L.A.C.S., S.G.C., C.J.W., P.T., and Y.F.E. edited and revised the manuscript; and L.A.C.S., S.G.C., C.J.W., P.T., and Y.F.E. approved the final version of the manuscript.

## References

[phy214753-bib-0001] Bakke, J. L. , & Darvill, F. T. (1957). Chlorisondamine (ecolid) chloride in medical treatment of severe hypertension. Journal of the American Medical Association, 163, 429–436. 13398287 10.1001/jama.1957.02970410019007

[phy214753-bib-0002] Banek, C. T. , Gauthier, M. M. , Helden, D. A. V. , Fink, G. D. , & Osborn, J. W. (2019). Renal inflammation in DOCA‐salt hypertension. Hypertension, 73, 1079–1086. 30879356 10.1161/HYPERTENSIONAHA.119.12762 PMC6540804

[phy214753-bib-0003] Barman, S. M. , & Yates, B. J. (2017). Deciphering the neural control of sympathetic nerve activity: Status report and directions for future research. Frontiers in Neuroscience, 11, 730. 29311801 10.3389/fnins.2017.00730 PMC5743742

[phy214753-bib-0004] Basting, T. , & Lazartigues, E. (2017). DOCA‐salt hypertension: An update. Current Hypertension Reports, 19, 32. 28353076 10.1007/s11906-017-0731-4 PMC6402842

[phy214753-bib-0005] Cerutti, C. , Barres, C. , & Paultre, C. (1994). Baroreflex modulation of blood pressure and heart rate variabilities in rats: assessment by spectral analysis. The American Journal of Physiology, 266, H1993–H2000. 8203598 10.1152/ajpheart.1994.266.5.H1993

[phy214753-bib-0006] Chadman, K. K. , & Woods, J. H. (2004). Cardiovascular effects of nicotine, chlorisondamine, and mecamylamine in the pigeon. Journal of Pharmacology and Experimental Therapeutics, 308, 73–78. 10.1124/jpet.103.057307 14566012

[phy214753-bib-0007] Constantinides, C. , Mean, R. , & Janssen, B. J. (2011). Effects of isoflurane anesthesia on the cardiovascular function of the C57BL/6 mouse. ILAR Journal, 52, e21–31. 21677360 PMC3508701

[phy214753-bib-0008] Esler, M. , Ferrier, C. , Lambert, G. , Eisenhofer, G. , Cox, H. , & Jennings, G. (1991). Biochemical evidence of sympathetic hyperactivity in human hypertension. Hypertension, 17, III29–III35. 2013490 10.1161/01.hyp.17.4_suppl.iii29

[phy214753-bib-0009] Fisher, J. P. , & Paton, J. F. (2012). The sympathetic nervous system and blood pressure in humans: Implications for hypertension. Journal of Human Hypertension, 26, 463–475. 21734720 10.1038/jhh.2011.66

[phy214753-bib-0010] Grassi, G. , Mark, A. , & Esler, M. (2015). The sympathetic nervous system alterations in human hypertension. Circulation Research, 116, 976–990. 25767284 10.1161/CIRCRESAHA.116.303604 PMC4367954

[phy214753-bib-0011] Grimson, K. S. , Tarazi, A. K. , & Frazer, Jr ., J. W. (1955). A new orally active quaternary ammonium, ganglion blocking drug capable of reducing blood pressure, Su‐3088. Circulation, 11, 733–741. 14364747 10.1161/01.cir.11.5.733

[phy214753-bib-0012] Huang, B. S. , Huang, X. , Harmsen, E. , & Leenen, F. H. (1994). Chronic central versus peripheral ouabain, blood pressure, and sympathetic activity in rats. Hypertension, 23, 1087–1090. 7911450 10.1161/01.hyp.23.6.1087

[phy214753-bib-0013] Lamprea‐Montealegre, J. A. , Zelnick, L. R. , Hall, Y. N. , Bansal, N. , & Boer, I. H. (2018). Prevalence of hypertension and cardiovascular risk according to blood pressure thresholds used for diagnosis. Hypertension, 72, 602–609. 30354757 10.1161/HYPERTENSIONAHA.118.11609 PMC6205214

[phy214753-bib-0014] Lerman, L. O. , Kurtz, T. W. , Touyz, R. M. , Ellison, D. H. , Chade, A. R. , Crowley, S. D. , Mattson, D. L. , Mullins, J. J. , Osborn, J. , Eirin, A. , Reckelhoff, J. F. , Iadecola, C. , & Coffman, T. M. (2019). Animal models of hypertension: A scientific statement from the American heart association. Hypertension, 73, e87–e120. 30866654 10.1161/HYP.0000000000000090 PMC6740245

[phy214753-bib-0015] Li, W. , Peng, H. , Cao, T. , Sato, R. , McDaniels, S. J. , Kobori, H. , Navar, L. G. , & Feng, Y. (2012). Brain‐targeted (pro)renin receptor knockdown attenuates angiotensin II–dependent hypertension. Hypertension, 59, 1188–1194. 22526255 10.1161/HYPERTENSIONAHA.111.190108 PMC3375126

[phy214753-bib-0016] Li, W. , Peng, H. , Mehaffey, E. P. , Kimball, C. D. , Grobe, J. L. , van Gool, J. M. , Sullivan, M. N. , Earley, S. , Danser, A. H. , Ichihara, A. , & Feng, Y. (2014). Neuron‐specific (pro)renin receptor knockout prevents the development of salt‐sensitive hypertension. Hypertension, 63, 316–323. 24246383 10.1161/HYPERTENSIONAHA.113.02041 PMC3947277

[phy214753-bib-0017] Li, W. , Sullivan, M. N. , Zhang, S. , Worker, C. J. , Xiong, Z. , Speth, R. C. , & Feng, Y. (2015). Intracerebroventricular infusion of the (Pro)renin receptor antagonist PRO20 attenuates deoxycorticosterone acetate‐salt‐induced hypertension. Hypertension, 65, 352–361. 25421983 10.1161/HYPERTENSIONAHA.114.04458 PMC4902274

[phy214753-bib-0018] Lu, Y. , Ma, X. , Sabharwal, R. , Snitsarev, V. , Morgan, D. , Rahmouni, K. , Drummond, H. A. , Whiteis, C. A. , Costa, V. , Price, M. , Benson, C. , Welsh, M. J. , Chapleau, M. W. , & Abboud, F. M. (2009). The ion channel ASIC2 is required for baroreceptor and autonomic control of the circulation. Neuron, 64, 885–897. 20064394 10.1016/j.neuron.2009.11.007 PMC2807410

[phy214753-bib-0019] Mancia, G. , & Grassi, G. (2014). The autonomic nervous system and hypertension. Circulation Research, 114, 1804–1814. 24855203 10.1161/CIRCRESAHA.114.302524

[phy214753-bib-0020] Marina, N. , Christie, I. N. , Korsak, A. , Doronin, M. , Brazhe, A. , Hosford, P. S. , Wells, J. A. , Sheikhbahaei, S. , Humoud, I. , Paton, J. F. R. , Lythgoe, M. F. , Semyanov, A. , Kasparov, S. , & Gourine, A. V. (2020). Astrocytes monitor cerebral perfusion and control systemic circulation to maintain brain blood flow. Nature Communications, 11, 131. 10.1038/s41467-019-13956-y PMC6952443 31919423

[phy214753-bib-0021] Maxwell, R. A. , Plummer, A. J. , & Osborne, M. W. (1956). Studies with the ganglionic blocking agent, chlorisondamine chloride in unanesthetized and anesthetized dogs. Circulation Research, 4, 276–281. 13317018 10.1161/01.res.4.3.276

[phy214753-bib-0022] Mayet, J. , & Hughes, A. (2003). Cardiac and vascular pathophysiology in hypertension. Heart, 89, 1104–1109. 12923045 10.1136/heart.89.9.1104 PMC1767863

[phy214753-bib-0023] Nakata, T. , Berard, W. , Kogosov, E. , & Alexander, N. (1991). Hypothalamic NE release and cardiovascular response to NaCl in sinoaortic‐denervated rats. American Journal of Physiology, 260, R733–738. 10.1152/ajpregu.1991.260.4.R733 1672796

[phy214753-bib-0024] Nomura, K. , Hiyama, T. Y. , Sakuta, H. , Matsuda, T. , Lin, C. H. , Kobayashi, K. , Kobayashi, K. , Kuwaki, T. , Takahashi, K. , Matsui, S. , & Noda, M. (2019). [Na(+)] increases in body fluids sensed by central nax induce sympathetically mediated blood pressure elevations via H(+)‐dependent activation of ASIC1a. Neuron, 101, 60–75.e66. 30503172 10.1016/j.neuron.2018.11.017

[phy214753-bib-0025] Nunes, F. C. , & Braga, V. A. (2011). Chronic angiotensin II infusion modulates angiotensin II type I receptor expression in the subfornical organ and the rostral ventrolateral medulla in hypertensive rats. Journal of the renin‐angiotensin‐aldosterone System, 12, 440–445. 21393361 10.1177/1470320310394891

[phy214753-bib-0026] Ohman, L. E. , Shade, R. E. , & Haywood, J. R. (1990). Parabrachial nucleus modulation of vasopressin release. American Journal of Physiology‐Regulatory, Integrative and Comparative Physiology, 258, R358–R364. 10.1152/ajpregu.1990.258.2.R358 2309929

[phy214753-bib-0027] Page, I. H. , & Olmsted, F. (1963). Hemodynamic mechanisms of increased cardiovascular response resulting from ganglioplegics and atropine. American Journal of Physiology‐Legacy Content, 204, 582–590. 10.1152/ajplegacy.1963.204.4.582 13941059

[phy214753-bib-0028] Ritchey, M. D. , Wall, H. K. , Owens, P. L. , & Wright, J. S. (2018). Vital signs: State‐level variation in nonfatal and fatal cardiovascular events targeted for prevention by million hearts 2022. Morbidity and Mortality Weekly Report, 67, 974–982. 30188881 10.15585/mmwr.mm6735a3 PMC6132183

[phy214753-bib-0029] Sabharwal, R. , Rasmussen, L. , Sluka, K. A. , & Chapleau, M. W. (2016). Exercise prevents development of autonomic dysregulation and hyperalgesia in a mouse model of chronic muscle pain. Pain, 157, 387–398. 26313406 10.1097/j.pain.0000000000000330 PMC4724275

[phy214753-bib-0030] Santajuliana, D. , Hornfeldt, B. J. , & Osborn, J. W. (1996). Use of ganglionic blockers to assess neurogenic pressor activity in conscious rats. Journal of Pharmacological and Toxicological Methods, 35, 45–54. 8645881 10.1016/1056-8719(95)00132-8

[phy214753-bib-0031] Schiltz, J. C. , Hoffman, G. E. , Stricker, E. M. , & Sved, A. F. (1997). Decreases in arterial pressure activate oxytocin neurons in conscious rats. American Journal of Physiology‐Regulatory, Integrative and Comparative Physiology, 273, R1474–R1483. 10.1152/ajpregu.1997.273.4.R1474 9362314

[phy214753-bib-0032] Seagard, J. L. , Elegbe, E. O. , Hopp, F. A. , Bosnjak, Z. J. , von Colditz, J. H. , Kalbfleisch, J. H. , & Kampine, J. P. (1983). Effects of isoflurane on the baroreceptor reflex. Anesthesiology, 59, 511–520. 6650907 10.1097/00000542-198312000-00005

[phy214753-bib-0033] Souza, L. A. C. , Worker, C. J. , Li, W. , Trebak, F. , Watkins, T. , Gayban, A. J. B. , Yamasaki, E. , Cooper, S. G. , Drumm, B. T. , & Feng, Y. (2019). (Pro)renin receptor knockdown in the paraventricular nucleus of the hypothalamus attenuates hypertension development and AT1 receptor‐mediated calcium events. American Journal of Physiology. Heart and Circulatory Physiology, 316, H1389–h1405. 30925093 10.1152/ajpheart.00780.2018 PMC6620680

[phy214753-bib-0034] Stocker, S. D. , Sved, A. F. , & Stricker, E. M. (2000). Role of renin‐angiotensin system in hypotension‐evoked thirst: studies with hydralazine. American Journal of Physiology‐Regulatory, Integrative and Comparative Physiology, 279, R576–R585. 10.1152/ajpregu.2000.279.2.R576 10938248

[phy214753-bib-0035] Thibodeau, J. F. , Holterman, C. E. , Burger, D. , Read, N. C. , Reudelhuber, T. L. , & Kennedy, C. R. (2014). A novel mouse model of advanced diabetic kidney disease. PLoS One, 9, e113459. 25514595 10.1371/journal.pone.0113459 PMC4267730

[phy214753-bib-0036] Trapold Joseph, H. , & Sullivan, J. G. (1956). Effect of ganglionic blocking agents upon blood flow and resistance in the superior mesenteric artery of the dog. Circulation Research, 4, 718–723. 13365082 10.1161/01.res.4.6.718

[phy214753-bib-0037] Trapold Joseph, H. , Sullivan Joan, G. , & Hawkins, J. L. (1957). Role of venous return in the cardiovascular response following injection of ganglion‐blocking agents. Circulation Research, 5, 444–450. 13447192 10.1161/01.res.5.4.444

[phy214753-bib-0038] Trebak, F. , Li, W. , & Feng, Y. (2018). Neuronal (pro)renin receptor regulates deoxycorticosterone‐induced sodium intake. Physiological Genomics, 50, 904–912. 30142028 10.1152/physiolgenomics.00065.2018 PMC6230870

[phy214753-bib-0039] Whelton, P. K. , Carey, R. M. , Aronow, W. S. , Casey, D. E. , Collins, K. J. , Himmelfarb, C. D. , DePalma, S. M. , Gidding, S. , Jamerson, K. A. , Jones, D. W. , MacLaughlin, E. J. , Muntner, P. , Ovbiagele, B. , Smith, S. C. , Spencer, C. C. , Stafford, R. S. , Taler, S. J. , Thomas, R. J. , Williams, K. A. , … Wright, J. T. (2018). 2017 ACC/AHA/AAPA/ABC/ACPM/AGS/APhA/ASH/ASPC/NMA/PCNA guideline for the prevention, detection, evaluation, and management of high blood pressure in adults: Executive summary: A report of the American College of Cardiology/American Heart Association Task Force on Clinical Practice Guidelines. Hypertension, 71, 1269–1324. 29133354 10.1161/HYP.0000000000000066

[phy214753-bib-0040] Xu, C. , Lu, A. , Lu, X. , Zhang, L. , Fang, H. , Zhou, L. , & Yang, T. (2017). Activation of renal (pro)renin receptor contributes to high fructose‐induced salt sensitivity. Hypertension, 69(2), 339–348. 27993957 10.1161/HYPERTENSIONAHA.116.08240 PMC5556690

[phy214753-bib-0041] Xu, J. , Molinas, A. J. R. , Mukerjee, S. , Morgan, D. A. , Rahmouni, K. , Zsombok, A. , & Lazartigues, E. (2019). Activation of ADAM17 (a disintegrin and metalloprotease 17) on glutamatergic neurons selectively promotes sympathoexcitation. Hypertension, 73(6), 1266–1274. 31006330 10.1161/HYPERTENSIONAHA.119.12832 PMC6506373

[phy214753-bib-0042] Xu, J. , Sriramula, S. , Xia, H. , Moreno‐Walton, L. , Culicchia, F. , Domenig, O. , Poglitsch, M. , & Lazartigues, E. (2017). Clinical relevance and role of neuronal AT_1_ receptors in ADAM17‐mediated ACE2 shedding in neurogenic hypertension novelty and significance. Circulation Research, 121, 43–55. 28512108 10.1161/CIRCRESAHA.116.310509 PMC5507353

[phy214753-bib-0043] Yin, F. C. , Spurgeon, H. A. , Rakusan, K. , Weisfeldt, M. L. , & Lakatta, E. G. (1982). Use of tibial length to quantify cardiac hypertrophy: Application in the aging rat. American Journal of Physiology, 243, H941–947. 10.1152/ajpheart.1982.243.6.H941 6216817

